# Distinct Ecologically Relevant Strains of *Anaplasma phagocytophilum*

**DOI:** 10.3201/eid1505.081502

**Published:** 2009-05

**Authors:** Janet E. Foley, Nathan C. Nieto, Robert Massung, Anthony Barbet, John Madigan, Richard N. Brown

**Affiliations:** University of California, Davis, California, USA (J.E. Foley, N.C. Nieto, J. Madigan); Centers for Disease Control and Prevention, Atlanta, Georgia, USA (R. Massung); University of Florida, Gainesville, Florida, USA (A. Barbet); Humboldt State University, Arcata, California, USA (R.N. Brown)

**Keywords:** Zoonoses, Anaplasma phagocytophilum, livestock, horses, woodrats, humans, letter

**To the Editor:** *Anaplasma phagocytophilum* was defined to include *Ehrlichia phagocytophila*, *E. equi*, and the agent of human granulocytic ehrlichiosis*.* Nevertheless, we and others have found phenotypic and genetic differences from diverse regions and hosts and conclude preliminarily that ecologically separate strains might exist that should be distinguished. Two precedents include ruminant strains of *A. phagocytophilum* in Europe and the Ap-Variant 1 from ruminants and ticks of North America and Europe.

In Europe, *A. phagocytophilum* infects livestock, rodents, and humans, with some species such as European cattle showing severe disease and high antibody prevalence. In contrast, cattle infection is rare in the United States, despite being common in other species. Experimental infection of cattle with California equine–origin strain MRK failed to induce disease or marked rickettsemia (*1*). Thus, even though European strains have ruminant tropism, an equine strain does not.

Ap-Variant 1 is found in ticks and deer in North America. This strain is distinctive in the 16S rRNA, major surface protein 4 (*msp4*)*, msp2*, and *ankA* genes (*2*). Deer, goats, and tick-derived cell lines can be infected with Ap-Variant 1, but rodents cannot (*3*). Our recent data examining *A. phagocytophilum* in western North America show at least 2 phenotypes: strains originating from sciurids (chipmunks and tree squirrels) and strains from woodrats (the previously postulated reservoir). In a survey of 2,121 small mammals in areas of California with enzootic Ap-Variant 1, seroprevalence was highest in tree squirrels (71%), woodrats (50%), and chipmunks (up to 28%), and PCR prevalence was highest in tree squirrels (16%) and chipmunks (34%) (*4*). We showed that chipmunks were competent reservoirs for *A. phagocytophilum* through exposure in the field, successful inoculation with strain MRK, and transmission through *Ixodes pacificus* to mice. However, discrepancy in the phenotype of strains originating from woodrats and chipmunks is substantial when these strains are inoculated into horses. One chipmunk strain can infect both rodents and horses (important laboratory animal models for human infection), whereas woodrat strains show restricted rodent-only tropism.

A naturally infected redwood chipmunk was trapped in Mendocino County, California, exsanguinated, and documented to be positive for *A. phagocytophilum* by using real-time PCR, with a cycle threshold (C_t_) of 31.31. An adult horse was negative for infection and exposure by PCR and immunofluorescence assay, premedicated with flunixin meglumine and diphenhydramine, and inoculated with 1.5 mL of infected chipmunk whole blood in EDTA. The mare was monitored daily for 16 days, including blood smear, serologic testing, and PCR, and assessment of behavior and attitude, rectal temperature, and legs for edema, swelling, or pain. She became ill 12 days postinoculation, with a body temperature of 40.0ºC, lethargy, depression, and inappetance. Blood smears showed *A. phagocytophilum* morulae in neutrophils, and she was PCR positive on day 13 (C_t_ 37). Thus, infection from this chipmunk strain was indistinguishable from that induced when human-origin *A. phagocytophilum* was inoculated into this horse. The mare recovered after treatment.

In contrast, woodrat strains show rodent-host tropism but are not infectious to horses. We attempted to infect 3 horses with *A. phagocytophilum* from naturally infected, PCR-positive woodrats from Hoopa Valley, Humboldt County (1 pool of 4, 1 single) and Henry Cowell State Park, Santa Cruz County (N = 1); both sites are enzootic for *A. phagocytophilum*. Woodrats were bled into tubes containing EDTA, and blood was kept cool and screened that day by real-time PCR and serologic testing. The PCR-positive samples were divided into rodent and horse inocula. The horses were negative for infection and exposure using PCR and immunofluorescence assay, premedicated as described above, and then inoculated with 6 mL of *A. phagocytophilum*–infected woodrat blood. These horses never became infected on the basis of clinical signs, serology, blood smears, and PCR. Each horse was reinoculated 1–2 months later with 1.5 mL of an equine-tropic strain (MRK or chipmunk) to verify susceptibility to infection. All 3 became ill within 12–13 days postinoculation, with substantial increase in body temperature (>39.4º C), lethargy, depression, and inappetance. Blood smears showed *A. phagocytophilum* morulae in neutrophils, and the animals were PCR positive and seroconverted. We considered the woodrat inocula unlikely to be noninfectious because aliquots of the same samples produced rickettsemia according to PCR in C3H/HeJ mice and uninfected woodrats. All 3 horses recovered after treatment.

Although some woodrat *A. phagocytophilum* strains are genetically similar to human and equine strains, others differ from sciurid, human, horse, and dog strains, with conserved blocks within the *msp2* gene (*5*). A phylogenetic tree based on the omp1n gene clusters a sciurid strain with local horses, distinct from northern California woodrats (Appendix Figure). Some woodrat strains have rodent-only tropism; Ap-Variant 1 does not infect rodents. Strains from sciurids and white-footed mice infect multiple laboratory animals and perhaps humans as well. Thus, epidemiologic studies evaluating human risk need to incorporate these distinctions and further ecologic and molecular genetic studies are necessary. With increasing reports of dissimilar genotypes of *A. phagocytophilum* from multiple regions of the world, defining distinct phenotypes and using nomenclature that appropriately clarifies the distinctions are important.

## Supplementary Material

Appendix Figure Bayesian phylogeny of the omp1n gene of A. phagocytophilum, with 100,000 iterations. Taxa are 2 woodrats from northern California, the human strain HZ, the California horse strain MRK, and a squirrel from Santa Cruz, CA. Scale bar indicates number of nucleotide substitutions per site.
