# Supplementary figures and images for: Distinct Ecologically Relevant Strains of Anaplasma phagocytophilum
Source: Emerg Infect Dis. 2009 May;15(5):842–3. doi: 10.3201/eid1505.081502 (PMC2687023; doi:10.3201/eid1505.081502)

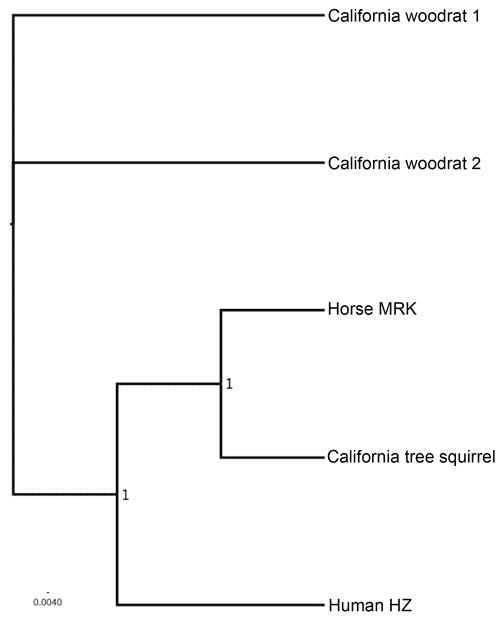

Supplement: Appendix Figure — Bayesian phylogeny of the omp1n gene of A. phagocytophilum, with 100,000 iterations. Taxa are 2 woodrats from northern California, the human strain HZ, the California horse strain MRK, and a squirrel from Santa Cruz, CA. Scale bar indicates number of nucleotide substitutions per site. [file 08-1502_app-s1.gif]
